# A Review of Cybersecurity Issues in Smart Meter-Based Energy Trading

**DOI:** 10.3390/s26123621

**Published:** 2026-06-06

**Authors:** Xingyu Yang, Hui Cui

**Affiliations:** Department of Software Systems and Cybersecurity, Faculty of IT, Monash University, Clayton Campus, Melbourne, VIC 3800, Australia; gelen.yang@monash.edu

**Keywords:** smart meters, advanced metering infrastructure, smart meter-derived records, local energy trading, transactive energy, peer-to-peer energy trading, security and privacy, data trust

## Abstract

Smart meters increasingly operate as grid-edge sensing and communication nodes, extending their role beyond conventional digital billing by generating records for local energy trading. In such settings, smart meter-derived records may support coordination, participant interaction, validation, billing, and settlement across different trading architectures. Once these records leave the metering edge, their security and privacy risks depend on how they are routed, reused, protected, and interpreted across centralized, transactive, and peer-to-peer trading workflows. In this review, we examine smart meter-based energy trading through a record-centric and framework-oriented lens. We first clarify the role of smart meters and smart meter-derived records, then compare three representative trading frameworks in terms of data-path structure, coordination pattern, trust organization, and validation or settlement positioning. Building on the comparison, we identify three lifecycle-based layers of issues: record integrity and temporal consistency, insecure transmission and interface access security, and confidentiality and privacy exposure. We also review existing mitigation mechanisms and remaining limitations for each issue layer. We conclude that future work should prioritize lifecycle-wide record governance, temporal continuity, privacy–accountability co-design, and deployable protection across hybrid trading environments.

## 1. Introduction

In smart meter-based energy trading, measurements generated at the metering edge become trading-relevant records for coordination, validation, billing, and settlement. Smart meters therefore extend beyond conventional digital billing: they act as grid-edge sensing and communication nodes that generate interval consumption and generation measurements, bidirectional import and export values, and other time-referenced observations [1,2,3,4]. Thus, the smart meter serves as both a metering endpoint and the sensing–communication origin of records later reused in trading workflows.

When smart meter-derived records are used beyond traditional billing, security and privacy concerns depend on both the metering source and the movement of records across trading arrangements. Recent literature shows that smart meter-based energy trading spans several distinct forms—in particular, traditional centralized energy trading [5,6], transactive energy [7,8], and peer-to-peer (P2P) trading [7,9]. These forms differ in coordination logic, participant interaction, and trust structure. As trading arrangements become more distributed and participant-facing, smart meter-derived records pass through additional interfaces, intermediaries, and less unified trust domains. Recent work on smart-meter data analytics and local energy communities also shows that these records are becoming operationally and market-relevant beyond conventional metering functions [3,10]. These conditions create risks related to record integrity, temporal consistency, interface security, privacy exposure, and settlement interpretation.

Existing reviews have already advanced understanding of several adjacent areas. These include local electricity market design [5]; the conceptual boundaries among peer-to-peer trading, transactive energy, and community self-consumption [7]; recent developments in transactive energy systems [8]; smart-meter data analytics for distribution-network applications [2]; AMI privacy and security [11]; smart-meter privacy-preserving techniques [12]; smart-grid privacy-preserving technologies [13]; and cybersecurity concerns in transactive energy markets [14]. These reviews provide important foundations for the understanding of market structures, enabling technologies, data analytics, privacy risks, and selected cybersecurity concerns.

However, existing review papers have not fully integrated how the same smart meter-derived record is formed, transmitted, admitted, validated, reused, and retained across smart meter-based energy trading workflows. This leaves the connections between source-side metering conditions and later cybersecurity and privacy consequences for validation, billing, settlement, and audit-related reuse insufficiently developed. To bridge this gap, this review follows smart meter-derived records across their lifecycle and analyses how their movement through these trading settings shapes cybersecurity and privacy issues.

In Table 1, we compare the present review with representative existing reviews in adjacent areas.

The contribution of this review is threefold. First, it uses smart meter-derived records as the common analytical object connecting metering, communication, trading architecture, validation, billing, settlement, and privacy exposure. Second, it compares centralized trading, transactive energy, and peer-to-peer trading according to how these records are routed, reused, exposed, and validated under different coordination and trust structures. Third, it maps cybersecurity issues and mitigation limitations onto the record lifecycle, showing where stage-specific protections still leave gaps in record admissibility, temporal validity, privacy–accountability co-design, and deployability. The novelty of the review is therefore synthetic and framework-oriented: it provides a record-lifecycle basis for analyzing cybersecurity in smart meter-based energy trading and integrates device security, communication security, privacy protection, and market settlement within a common analytical framework.

The review focuses on three issue layers: record integrity and temporal consistency, transmission and interface security, and confidentiality and privacy exposure. Broader issues such as wholesale market design, regulatory reform, and general pricing strategies are considered only when they directly affect smart meter-derived records.

### Organization

The remainder of this review is organized as follows. Section 2 defines the review scope, methodology, search terms, and paper-selection logic. Section 3 introduces smart meter-derived records and compares centralized trading, transactive energy, peer-to-peer trading, and hybrid or multi-framework studies. Section 4 introduces the attacker model and discusses three cybersecurity issue layers: record integrity and temporal consistency, insecure transmission and interface access, and confidentiality and privacy exposure. Section 5 reviews existing mitigation mechanisms and summarizes their limitations. Section 6 identifies future research directions for record admissibility, temporal continuity, privacy–accountability co-design, and deployable protection across hybrid trading environments. Section 7 concludes the review.

## 2. Review Scope and Methodology

This review adopts a structured, topic-led search-and-selection procedure to support a framework-oriented synthesis of cybersecurity issues in smart meter-based energy trading. The review focuses on studies that explain how smart meter-derived records are generated, transmitted, validated, protected, and reused across energy trading workflows. The evidence base was developed through targeted literature searches, publisher-source checking, and backward–forward reference tracing. Searches were conducted through academic search tools, publisher platforms, DOI records, and technical-standard sources relevant to smart metering, energy trading, and smart-grid cybersecurity. Representative sources included Google Scholar, IEEE Xplore, ScienceDirect, SpringerLink, MDPI, Wiley/IET, IEC, and NIST. Search terms combined concepts related to smart meters, advanced metering infrastructure (AMI), local energy markets, transactive energy, peer-to-peer energy trading, data integrity, false data injection, replay attacks, authentication, secure communication, privacy, confidentiality, billing, settlement, blockchain, and smart contracts.

Studies were included when they addressed cybersecurity, privacy, or trust issues in smart meters, AMI, or smart meter-derived records; centralized, transactive, or peer-to-peer energy trading frameworks; mitigation mechanisms for integrity, temporal consistency, secure transmission, authentication, confidentiality, privacy protection, billing, or settlement; or architectural and survey-based insights into record exchange and reuse in local energy markets. Priority was given to recent peer-reviewed studies, especially those published from 2021 onward, where the role of metering data, communication paths, trading coordination, validation, billing, or settlement was clearly described. Earlier foundational studies, standards, and technical reports were retained where they supported key definitions, threat categories, communication-security requirements, or privacy risks. Studies were excluded when they focused on generic smart grid control, generic blockchain or cryptographic techniques, or broad electricity-market design without a clear connection to smart meter-derived records or trading workflows.

Search results and reference-tracing candidates were screened by title, abstract, and keywords, followed by full-text evaluation. The selected studies were classified according to trading framework, security issue, mitigation approach, and remaining limitations. Traditional centralized energy trading, transactive energy, and peer-to-peer energy trading were used as the three main comparative settings because they differ in data-path structure, coordination logic, trust organization, and validation or settlement positioning. Studies that combine or compare two or more of these settings were classified separately as hybrid or multi-framework studies. The framework-specific tables serve as structured comparison tools for the examination of how cybersecurity and privacy issues change as smart meter-derived records move from measurement and reporting to communication, coordination, validation, billing, and settlement.

## 3. Preliminaries

In this section, we provide an overview of relevant definitions and concepts to be used in this review paper.

### 3.1. Smart Meters in Energy Trading

Smart meters are digital metering devices installed at the grid edge to measure consumption, generation, import, and export at fine temporal intervals [1,2]. Through advanced metering infrastructure (AMI), these measurements are communicated to utility, aggregator, or platform-side systems [1,3]. In prosumer-oriented settings, they distinguish local surplus from deficit and can support aggregation, coordination, matching, validation, billing, and settlement in transactive and peer-to-peer trading [7,8,9,15].

This review treats smart meter-derived records as the main analytical object and the smart meter as the source device. A smart meter-derived record refers to participant-level metering outputs that later enter trading workflows, including interval measurements of electricity consumption and generation, bidirectional import and export values, and other time-referenced observations extracted from smart metering infrastructure [1,2,3,16]. These records originate at the metering edge and later enter trading and settlement workflows, extending their role beyond measurement alone.

For analytical clarity, this review uses five analytical lifecycle forms to describe how smart meter-derived records are transformed, checked, reused, or retained across energy trading workflows. These forms build on smart-meter data definitions and data-metering exchange standards, which define the measurement origin and data-exchange basis of smart meter-derived records [1,16]. They also reflect how meter-originated data are reused in energy trading, validation, billing, settlement, and accountability processes [7,9,17,18,19]. These forms are not separate data sources. They describe how a meter-originated measurement is selected, processed, validated, reused, or retained as it moves through an energy trading workflow. In the remainder of this review, “smart meter-derived records” is used as an umbrella term for these related lifecycle forms unless a specific lifecycle form is being discussed. Table 2 summarizes these forms and their cybersecurity relevance.

### 3.2. An Overview of Energy Trading in Smart Metering Systems

Building on the record-centric definition, this review treats energy trading in smart metering systems as a record-dependent workflow shaped by local market mechanisms and trading architectures [5,22]. In this workflow, smart meter-derived records support coordination, matching, validation, billing, and settlement after they leave the metering edge [7,8,9].

Based on how these records are handled after measurement, this review compares three analytical reference points: traditional centralized trading [6,23,24], transactive energy [8,25,26], and peer-to-peer (P2P) trading [9,27,28]. The categories are compared by dominant record path, coordination pattern, trust organization, interface exposure, and validation or settlement positioning. Practical systems may combine these properties. A P2P market may still use platform-based clearing, billing, blockchain, or smart-contract support, while a transactive energy system may include local participant-facing exchanges [7,29]. Section 3.6 discusses studies that explicitly combine or compare two or more of these frameworks.

Table 3 summarizes the main architectural differences among centralized, transactive, and peer-to-peer energy trading, with attention to record positioning, interface exposure, validation responsibility, privacy risk, and settlement-related dispute risk after the metering edge.

**Figure 1:**
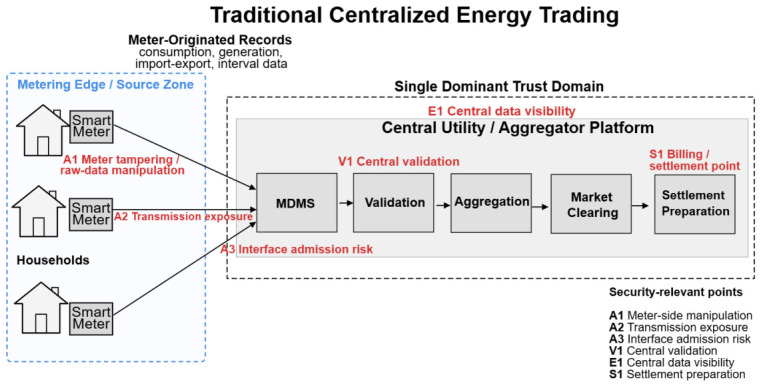
Traditional centralized energy trading architecture with cybersecurity-relevant workflow points. Smart meter-derived records mainly follow a platform-facing path from the metering edge to a central utility or aggregator platform. The figure highlights meter-side manipulation, transmission exposure, interface-admission risk, central validation, central data visibility, and billing or settlement points.

**Figure 2:**
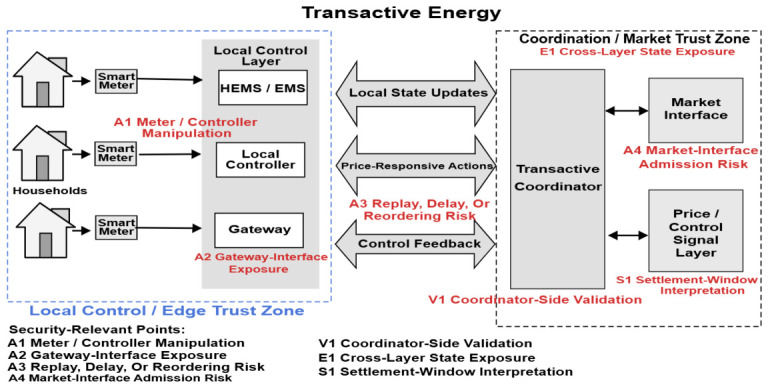
Transactive energy architecture with cybersecurity-relevant workflow points. Smart meter-derived records and local state information are repeatedly reused across smart meters, local controllers, gateways, coordinators, and market-facing interfaces. The figure highlights meter and controller manipulation; gateway-interface exposure; replay, delay, or reordering risk in repeated coordination; market-interface admission risk; coordinator-side validation; cross-layer state exposure; and settlement-window interpretation.

**Figure 3:**
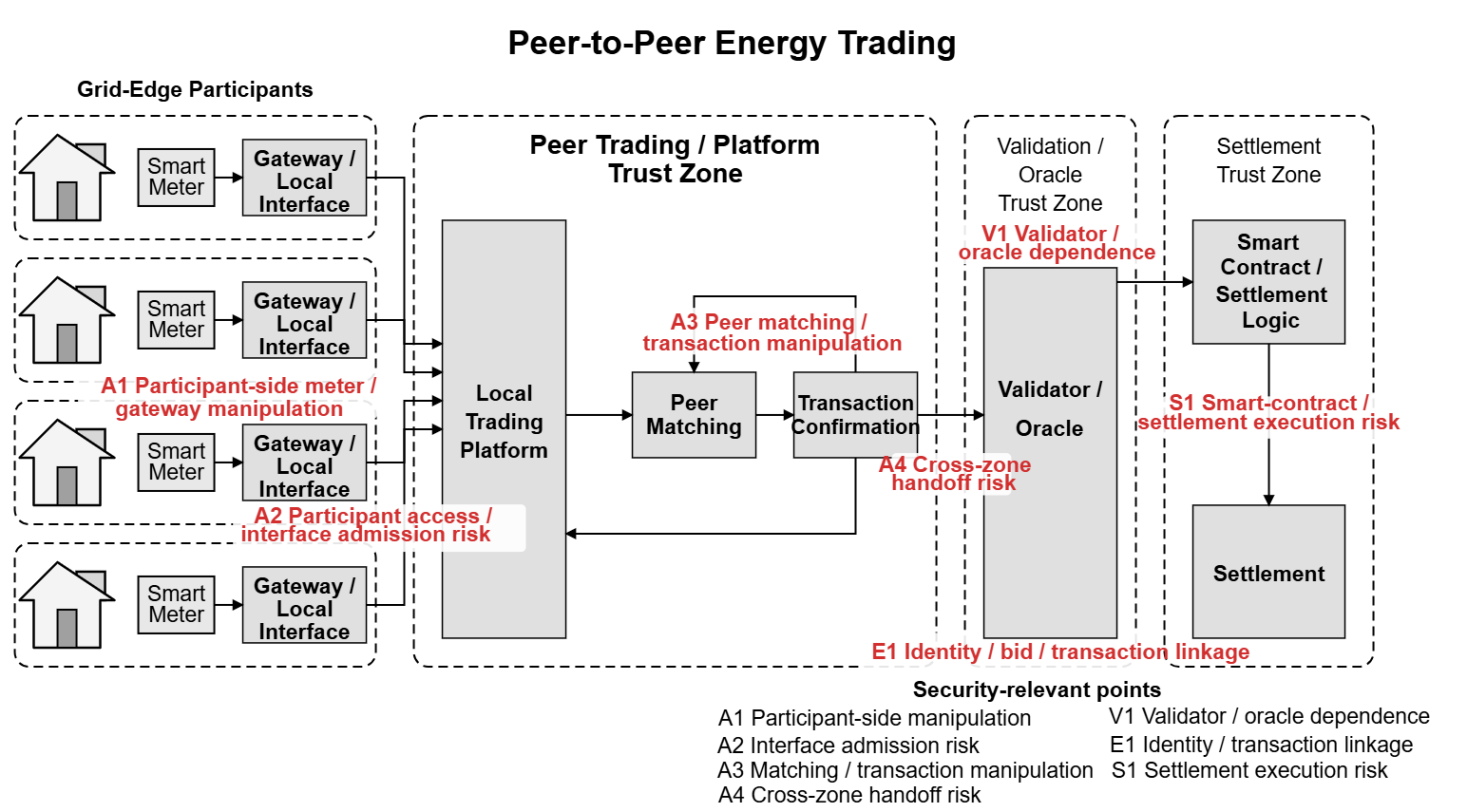
Peer-to-peer energy trading architecture with cybersecurity-relevant workflow points. Smart meter-derived records and trading inputs move from participant-side meters and gateways into peer-facing matching, transaction confirmation, validation or oracle services, and settlement logic. The figure highlights participant-side manipulation, interface-admission risk, peer-matching and transaction-manipulation risk, cross-zone handoff risk, validator or oracle dependence, identity and transaction linkage, and smart-contract or settlement-execution risk.

The following subsections first examine the three main trading mechanisms, then discuss studies that combine or compare two or more of them. For the three main mechanisms, the security-relevant labels in Figure 1, Figure 2 and Figure 3 connect the architectural comparison with the issue analysis in Section 4. They indicate where smart meter-derived records encounter source-side manipulation, transmission exposure, interface admission, validation dependence, data visibility, linkage, and settlement-related risks under centralized, transactive, and peer-to-peer trading arrangements. These labels mark architecture-specific workflow points through which the issue layers in Section 4 become visible.

### 3.3. Traditional Centralized Energy Trading

As illustrated in Figure 1, traditional centralized energy trading represents the most bounded data-path configuration among the three architectures considered in this review. Under this arrangement, smart meter-derived records are primarily collected at the grid edge and transmitted in a largely unidirectional manner toward a utility, aggregator, or other centrally governed platform where coordination, accounting, and settlement-related processing remain institutionally concentrated [6]. Community-based centralized market designs further show how auction or preference-based trading can still be organized through a platform-facing workflow [23]. In practice, this platform-side authority may be implemented through operator-facing infrastructure such as meter data management systems (MDMSs), community-level aggregators, or utility-controlled market interfaces [24].

Table 4 summarizes the selected centralized trading studies according to their main record-handling, coordination, and market-design features.

### 3.4. Transactive Energy

As illustrated in Figure 2, transactive energy introduces a coordination-intensive architecture in which smart meter-derived records circulate through local controllers, energy management systems, gateways, higher-layer coordinators, and market interfaces [8,25,26,43]. These records support iterative coordination through local state updates, price-responsive actions, and dispatch-related adjustments across multiple architectural layers [30,44].

Transactive energy depends on timely communication, interface consistency, and reliable state exchange because coordination decisions are repeatedly updated from local measurements and higher-layer responses [26,30,43,44]. It therefore forms a partially distributed trust structure between centralized platform control and more fragmented participant-facing P2P trading [7,8,25].

Table 5 summarizes the selected transactive energy studies according to their main record-handling, coordination, and deployment-related features.

### 3.5. Peer-to-Peer Energy Trading

As illustrated in Figure 3, peer-to-peer (P2P) energy trading represents the architecture in which smart meter-derived records become most directly participant-facing and are exposed to the most fragmented trust conditions among the three arrangements considered in this review. Unlike traditional centralized trading, where meter-originated data are largely absorbed into a utility- or platform-controlled workflow, P2P trading places greater emphasis on direct or semi-direct interaction among participants [7,9,45]. P2P market reviews further discuss enabling models, transactive settings, and implementation challenges for participant-facing energy trading [27,28,46,47]. Under this arrangement, smart meter-derived records are no longer mainly operator-facing inputs for centralized accounting or coordination. They become part of the data path that supports peer matching, transaction logic, validation, and settlement-related functions.

In P2P trading, smart meter-derived records move through participant-facing and multi-hop workflows after measurement. Trading-relevant meter data or abstractions derived from them may pass through gateways, local platforms, peer interfaces, transaction-confirmation mechanisms, validation or oracle services, and settlement layers before transaction closure. P2P sharing and flexibility-market studies show how participant-side inputs support local trading and coordination [31,48], while decentralized P2P designs illustrate distributed decision-making and privacy-aware validation conditions [33]. Because transaction confirmation, validation, and settlement form separate trust points, cross-zone handoff can create validation, accountability, and dispute-resolution risk.

Table 6 summarizes the selected peer-to-peer trading studies according to their main record-handling, transaction, validation, and settlement-related features.

### 3.6. Hybrid and Multi-Framework Energy Trading Studies

Several studies combine or compare two or more trading frameworks within the same review, design, or implementation setting. Capper et al. reviewed peer-to-peer trading, community self-consumption, and transactive energy as local energy market models, comparing participation structure, governance, topology, and market-design characteristics [52]. Their comparison shows how local energy-market models can overlap through participation rules, governance arrangements, and coordination mechanisms.

Centralized coordination also appears inside community-level or peer-facing markets. Goitia-Zabaleta et al. proposed a two-stage centralized management approach for a local energy market that integrates prosumers in a community-based P2P setting [6]. In this design, prosumer participation is organized through a centrally managed process involving energy planning, dispatch optimization, and real-time management. Gasca et al. examined fairness in energy communities by comparing centralized and decentralized organizational frameworks for operation and cost sharing [42]. These studies describe community trading arrangements that combine central coordination, shared-asset management, participant-level allocation, and decentralized participation within the same market setting.

P2P trading is also combined with transactive coordination. Xia et al. reviewed peer-to-peer transactive energy markets and discussed trading environments, market structures, market mechanisms, trading platforms, optimization methods, and distributed energy resources [28]. Ying et al. proposed decentralized energy management for a hybrid residential–commercial building cluster using peer-to-peer transactive energy trading [53]. Liaquat et al. proposed an integrated two-stage hybrid P2P–demand-response transactive energy trading platform that links demand-response scheduling with P2P trading through a distributed optimization process [54]. These works place participant-level exchange inside a broader coordination environment where local trading, flexibility scheduling, and system-level optimization are coupled.

Blockchain-oriented studies extend hybridization through shared coordination, validation, and settlement layers. Mazrae et al. reviewed blockchain-based transactive energy and peer-to-peer energy trading systems and proposed a generalized cyber-physical framework for blockchain-supported TE and P2P platforms [29]. Tooki et al. proposed an implementation framework for a decentralized peer-to-peer transactive energy system, linking decentralized P2P exchange with TES implementation [55]. Across these studies, blockchain and smart-contract mechanisms support transaction recording, validation, and settlement in mixed trading arrangements.

## 4. Security Issues in Smart Meter-Based Energy Trading

This section examines three cybersecurity issue layers in smart meter-based energy trading, consistent with broader smart-grid cybersecurity concerns around system characteristics, risks, vulnerabilities, and protection strategies [56]. Record integrity and temporal consistency address the accuracy and timing of smart meter-derived records. Insecure transmission and interface access security address in-transit protection and whether receiving interfaces admit only legitimate inputs. Confidentiality and privacy exposure address the disclosure of sensitive household behavior and the linkage of trading-relevant data across repeated trading stages.

These issue layers correspond to the main points where smart meter-derived records can be compromised: formation, transmission, interface admission, validation, and reuse. For each layer, this section examines how the issue appears across centralized, transactive, and peer-to-peer trading frameworks and how it affects coordination, validation, billing, and settlement. Other cyber, market, or operational risks are discussed only when they directly affect smart meter-derived records or trading-related data use.

Building on this workflow view, this review uses a workflow-based attacker model. The model treats attacker capabilities as stage-specific and potentially partial across record formation, transmission, interface admission, validation, reuse, and retention. Table 7 summarizes the attacker capabilities considered in this review and links them to the corresponding record lifecycle forms and security consequences.

### 4.1. Record Integrity and Temporal Consistency

In smart meter-based energy trading, source-side trustworthiness depends on whether meter-originated values remain accurate and temporally aligned with the reporting, coordination, and settlement intervals in which they are used. Trading, validation, billing, and settlement rely on values that correctly represent consumption, generation, import, export, or local energy exchange [20]. They also depend on correct timing, ordering, and synchronization across communication, coordination, and settlement processes [17,66].

Record integrity is compromised when the numerical content of a meter-originated record is altered before or during its reuse in trading workflows. At the metering edge, tampering may interfere with the sensing or metering process itself—for example, by bypassing part of the current path or manipulating the measurement chain—so that the generated record already departs from the underlying physical electricity activity [57,58]. False data injection introduces fabricated or strategically manipulated values into the measurement or reporting stream, where they may later be treated as valid trading inputs [63,67]. Unauthorized modification can also occur after generation, when reported import, export, consumption, or generation values are altered before aggregation, validation, or settlement reuse. These attacks can turn a source-side measurement into a distorted trading input, misrepresenting local surplus, deficit, or participant-level exchange and weakening later coordination, validation, billing, and settlement [20].

Temporal consistency may fail even when the recorded value, itself, is not falsified. Replay resends an outdated measurement as if it were current, while delay causes a valid record to arrive outside its intended update cycle or settlement window. Replacement allows one record to stand in for another, and reordering changes the processing sequence so that updates may be applied at the wrong stage or in the wrong order [17]. Timestamp manipulation shifts a record into an incorrect reporting interval without necessarily changing its numerical content, while clock desynchronization causes devices or coordination layers to disagree on when a measurement should be considered valid [66,68]. In this case, the security failure lies in the timing and sequencing of the record: a valid reading may become stale, miss its settlement window, or disrupt the order on which coordination decisions depend.

In energy trading, these timing failures change the market meaning of otherwise valid values. A replayed value may be reused in a later trading interval, and a delayed value may miss bidding, coordination, clearing, or settlement cutoffs [17,66]. Reordering can cause controllers, coordinators, or market platforms to apply updates in the wrong sequence, distorting price-responsive scheduling, matching, or dispatch-related decisions [17]. Timestamp manipulation can shift the same physical quantity into a different tariff, price, or trading interval, changing its financial meaning [17,21]. Clock desynchronization can weaken interval attribution and create mismatches between committed and delivered energy volumes, increasing billing-dispute and reconciliation pressure [18,21,66]. Temporal consistency is therefore a trading-specific trust condition linking measurement time with coordination, validation, billing, and settlement [21,65].

The impact of record-integrity attacks varies across trading frameworks. In traditional centralized trading, falsified records are typically absorbed into a relatively bounded platform- or aggregator-facing chain, where they mainly distort aggregation, validation, and settlement preparation on the operator side [20,67,69]. Transactive energy systems are more exposed to propagation effects because records are repeatedly exchanged across controllers, coordinators, and trading layers; once falsified values enter these loops, they can degrade matching, price-responsive control, scheduling, and dispatch-related decisions [17,30,70]. In peer-to-peer trading, integrity failures are harder to contain because records move through more distributed and participant-facing interactions, where malicious data injection or Byzantine manipulation may appear as inaccurate or untrusted trading input [64]. These failures may also surface as disputed validation, billing discrepancies, and settlement-reconciliation pressure, especially when committed and delivered energy volumes must be reconciled under accountability requirements [18,65].

These temporal failures play out differently across trading frameworks. In traditional centralized trading, replayed, delayed, or temporally misattributed records mainly affect a bounded validation and settlement chain because the platform may process a stale or wrongly timed record as valid for the current reporting interval [66]. In transactive energy systems, the effect is broader because coordination depends on repeated updates and correct sequencing across distributed control and market-facing layers. Replay, delay, replacement, reordering, or desynchronization can misalign local state estimation with higher-layer responses, reducing the reliability of iterative coordination and trading-related scheduling decisions [17,66,68]. In peer-to-peer trading, temporal misattribution is especially difficult to resolve after records have crossed multiple handoff points before validation and settlement closure. This creates uncertainty about the correct trading interval and increases pressure on participant-facing validation and billing reconciliation, especially when committed and delivered energy volumes must be matched over time [18]. It may also increase the need for secure pricing and settlement-support mechanisms in P2P trading [65].

### 4.2. Insecure Transmission and Interface Access Security

After a smart meter-derived record leaves the metering edge, its trustworthiness depends on the communication path and the interfaces through which it enters trading workflows. Insecure transmission involves interception, modification, delay, replay, or out-of-context delivery of records and trading messages across communication links [14,17]. Interface access security concerns whether gateways, controllers, coordinators, platforms, or application programming interfaces (APIs) accept only legitimate and authorized records, requests, or commands [60,61]. These risks can compromise a valid record after generation, either through in-transit manipulation or untrusted interface admission before coordination, validation, or settlement reuse [17,60,61].

Transmission exposure can occur between meters, gateways, controllers, coordinators, platforms, or participant-facing components. In a man-in-the-middle attack, an adversary interferes with message exchange between legitimate nodes without appearing as the original sender [59]. In-transit tampering modifies the content of a record or message before higher-layer reuse [59]. Replay resends a previously valid message in a later interaction context where it no longer belongs [17]. Insecure protocol implementation can further weaken message authenticity, confidentiality, or contextual validity during exchange [14,59]. As a result, records that were valid at origin may arrive altered, duplicated, delayed, or detached from the context in which they were meant to be used.

On the receiving side, gateway or interface compromise can allow an intermediary node to relay manipulated records or coordination messages. Device or interface impersonation allows an attacker to masquerade as a legitimate participant or infrastructure component, while spoofing falsifies the apparent identity or origin of the submitted input. AMI authentication and intrusion-detection studies treat these identity and admission risks as central interface-security problems [60]. Weak admission controls may allow false records, manipulated requests, or illegitimate coordination commands to enter the trading workflow. Authentication and key-agreement protocols address illegitimate session establishment and role misuse [61,62], while lightweight AMI security work addresses constrained interface protection [71]. The practical risk is that untrusted inputs may be admitted as legitimate trading data.

Transmission risks vary across trading frameworks because communication paths are not equally exposed. In traditional centralized trading, these risks are mainly concentrated along a bounded meter–gateway–platform or aggregator-facing chain [6], where delayed, lost, or manipulated records can contaminate centralized data ingestion, validation, and settlement preparation [34]. In transactive energy systems, repeated exchange among controllers, gateways, coordinators, and market-facing interfaces increases exposure to communication-layer attacks [14]. Message loss, tampering, replay, or reordering can propagate through coordination loops and misalign higher-layer responses with local state updates [17,72]. In peer-to-peer trading, participant-facing exchange and distributed validation create longer and more fragmented communication paths [9,29], increasing participant-side verification burden [33] and reconciliation pressure during billing or settlement [65].

Weak interface admission creates a related but distinct problem. In traditional centralized trading, unauthorized records, requests, or commands mainly enter a bounded platform- or aggregator-facing chain [6], compromising centralized validation and settlement preparation when interface admission or authentication is weak [60]. In transactive energy systems, spoofed, compromised, or unauthorized nodes can inject untrusted inputs into repeated controller–gateway–coordinator loops, where they may distort higher-layer control, matching, and trading decisions [14,72]. In peer-to-peer trading, unauthorized access is harder to contain because participant-facing exchange and distributed validation allow untrusted submissions to enter multi-hop workflows before settlement closure [9,29]. This increases exposure to disputed transactions and places a heavier burden on participant identity verification, counterparty validation, and settlement accountability [65].

### 4.3. Confidentiality and Privacy Exposure of Trading-Relevant Meter Data

In smart meter-based energy trading, confidentiality and privacy risks arise through two main channels: fine-grained meter-data exposure and trade-linkage risks involving identity disclosure. Fine-grained exposure occurs when trading-relevant records reveal household consumption, generation, occupancy, appliance use, or routine behavior. Trade-linkage risks arise when those records become associated with recurring participants, bids, trades, billing records, or settlement outcomes across trading workflows. These risks matter because electricity data can reveal behavioral patterns and household-level information [12,13]. Transactive energy increases the reuse of smart meter-derived records across repeated coordination and market-facing interactions [8]. Peer-to-peer trading further exposes meter-derived records to participant-facing validation, billing, and settlement contexts [9]. Blockchain-enabled TE and P2P designs can add further transaction-recording and settlement layers [29]. Privacy exposure therefore depends both on what smart meter-derived records reveal directly and on how they become linkable across later trading stages.

Fine-grained exposure becomes more severe when smart meter-derived records are collected, retained, or shared at a temporal and numerical resolution greater than market operation strictly requires. Electricity data have long been recognized as capable of disclosing household behavior, occupancy, and appliance-use characteristics [12,73]. This risk remains even when the data are not presented as explicit household profiles. Fine-grained consumption time series can be highly unique and support household re-identification from only a few consecutive measurements [74]. Half-hourly smart-meter data can also reproduce occupant behavior in residential energy analysis [75], and even very low-frequency readings may support appliance-level disaggregation through non-intrusive load monitoring [76]. Privacy loss therefore does not require direct disclosure of personal identifiers: consumption, generation, import, or export records may already be detailed enough to reveal household activity, energy-use patterns, and routine changes before they are reused in trading workflows.

Beyond fine-grained data exposure, privacy risk also accumulates when trading records are repeatedly linked to participant accounts, authentication exchanges, bids, trades, billing records, or settlement artifacts. In blockchain-based energy trading, repeated participant association and transaction linkage can expose trading behavior even when raw meter values are not directly disclosed [38]. On-chain transparency can reveal bidding price and quantity information in blockchain-based P2P electricity trading [40]. Public-blockchain transactional data may reveal prosumer energy profiles or allow third parties to infer load profiles unless these data are protected before trading and settlement reuse [39,50]. Smart contract-based energy trading further links transaction execution with trading and settlement processes, which can increase exposure when transactional data are not protected before reuse [35]. Privacy loss therefore accumulates through repeated market interactions that make records linkable to recurring participants, bidding behavior, and settlement outcomes.

The impact of fine-grained exposure varies across trading frameworks because visibility over smart meter-derived records is organized differently. In traditional centralized trading, exposure is concentrated within a relatively bounded utility-, aggregator-, or distribution system operator (DSO)-facing chain, so the main privacy concern is centralized visibility over detailed household production and consumption patterns [36]. In transactive energy systems, metering data and local state updates are reused across controllers, coordinators, and hierarchical market interactions [17]. This creates more opportunities for cross-layer behavioral inference when privacy-preserving coordination is not built into the trading process [37]. In peer-to-peer trading, participant-facing interaction and distributed market logic make meter-derived data or abstractions derived from them more informative about prosumer behavior. Some P2P designs use historical smart-meter readings to infer participant socio-demographic characteristics and feed those inferences back into market decision making [49]. As trading becomes more decentralized and participant-facing, fine-grained records become harder to contain as purely operational measurements.

Trade-linkage risks also vary across trading frameworks. In traditional centralized trading, association is mainly concentrated within a bounded operator-, aggregator-, or DSO-facing record chain, allowing a single authority to correlate metering, billing, and account information, even when the data are not disclosed to other participants [36]. In transactive energy systems, repeated coordination across controllers, coordinators, and hierarchical market interactions creates more opportunities to link local measurements with participant responses across stages [17,37]. In peer-to-peer trading, participant authentication and repeated transaction participation can associate users with trading activity [38]. Bidding-price and quantity information may become exposed during blockchain-based market clearing [40]. Public-chain transaction records and smart-contract execution can add further linkage risks if transactional data are not encrypted [39,50]. Billing and settlement artifacts may reinforce these associations over time [18]. Privacy risk therefore shifts from centralized record concentration toward cumulative linkage across market interactions, validation steps, billing records, and settlement outcomes.

## 5. Mitigation Directions and Remaining Limitations

This section reviews mitigation mechanisms for the cybersecurity issues identified in the previous section. It evaluates these approaches in terms of scalability; computational and communication overhead; deployment feasibility; and applicability to coordination, validation, billing, and settlement workflows.

### 5.1. Mitigating Record Integrity and Temporal-Consistency Risks

The first mitigation area corresponds to the source-side risks discussed in Section 4.1. Existing studies address this layer from two main perspectives: protecting the integrity of meter-originated records and preserving the temporal validity of records as they are exchanged and reused. These mechanisms reduce specific forms of tampering, false-data injection, replay, reordering, and synchronization-related disruption. Molina-Moreno et al. proposed a hardware-assisted anti-tampering mechanism based on embedded load injection, where a controlled resistive load is activated inside the smart meter to produce a known current increment. By comparing the measured current before and after injection, the meter verifies the integrity of its sensing path and detects partial bypass tampering in real time with minimal additional hardware [58]. For falsified records in operator-facing trading and scheduling chains, Kermani et al. introduced an XGBoost-assisted false-data detection and correction method for interconnected local energy networks, extending integrity protection to local trading and flexibility transactions [69]. Liu et al. addressed false data injection attacks (FDIAs) under load-aggregator interaction by jointly modeling and detecting attacks in a cyber-physical distribution setting so that falsified records can be identified before they are reused in aggregator-facing coordination [67]. Zhu et al. further developed a metering-platform detector that combines gradient lifting decision trees with multilayer perceptron (MLP) neural networks, showing that false-data manipulation can also be detected on the platform side after records enter automatic metering-data collection workflows [77]. In distributed peer-to-peer trading, Liu et al. proposed a Byzantine-resilient trading framework with online spatial–temporal anomaly detection to identify and mitigate malicious false-data manipulation during participant-level exchanges [64].

Temporal-consistency mechanisms address whether a valid record remains correctly ordered, attributed, and interpretable within the appropriate reporting or settlement interval. Lu et al. proposed a transactive energy system (TES) protection framework using enhanced Paillier encryption, digital signatures, and stamp concatenation [17]. The framework detects injected, replaced, and reordered records over insecure links, thereby protecting data confidentiality and the temporal ordering of reused records. Kumar et al. further addressed synchronization-targeted false-data attacks in networked microgrids by proposing a blockchain-enabled detection framework that uses synchronized micro-phasor measurement unit (µPMU) measurements and smart contract-based validation to secure voltage, phase-angle, and frequency synchronization across points of common coupling [68]. In the timing-protocol layer, Idrees et al. developed a lightweight attack-detection-and-mitigation framework for IEEE 1588 Precision Time Protocol (PTP) networks, where PTP is a clock-synchronization protocol for networked systems, aiming to improve timing-layer resilience without imposing excessive computational overhead on resource-constrained devices [78].

The main limitation is the fragmentation of protection across attack points, timing layers, and downstream reuse decisions. Existing mechanisms protect sensing-path integrity, false-data detection, message ordering, synchronization, and timing-protocol resilience at separate points [17,58,64,67,68,69,78]. Their deployment depends on meter-side support; representative training data; near-real-time processing; and additional communication, storage, or latency requirements. They provided limited guidance on how a challenged smart meter-derived record should remain admissible, temporally valid, or auditable during later coordination, validation, billing, and settlement reuse. Table 8 summarizes these mitigation directions.

### 5.2. Mitigating Transmission and Interface-Access Risks

The second mitigation area addresses the interaction-side risks discussed in Section 4.2: communication-channel protection, message authentication, device or participant verification, and interface-behavior detection. These mechanisms align with broader power-system communication-security concerns in the IEC 62351 series [79]. In the communication layer, Gormus et al. implemented OSCORE, an end-to-end security protocol for constrained CoAP communication, over 6TiSCH, a low-power multi-hop wireless framework for smart-grid AMI [80]. Ho et al. proposed a lightweight hybrid signcryption scheme, combining encryption and digital signing, for transmitted power-consumption data in resource-constrained smart-grid environments [81]. Nazir et al. combined additive secret sharing, MAC verification, and blockchain-enabled settlement to protect P2P energy and price information during transmission and transaction closure [65].

Transmission protection alone does not establish whether the submitting entity should be trusted. Authentication-oriented studies therefore focus on session establishment, device legitimacy, and runtime detection of spoofing or identity-based attacks. Cheng et al. proposed an anonymous, certificateless authentication and key-agreement scheme to strengthen mutual authentication and session-key establishment under smart-grid constraints [61]. Ponnuru et al. combined elliptic-curve cryptography, physical unclonable functions, and blockchain-assisted key management for device legitimacy checking in smart microgrids [62]. Achaal et al. proposed an intelligent authentication and intrusion detection system for AMI, extending protection from initial admission control to runtime checking against spoofing and identity-based attacks [60].

Transmission mechanisms remain subject to deployment and scalability constraints. Gormus et al. evaluated OSCORE over 6TiSCH in a controlled setting, leaving further testing under complex urban environments, dynamic topologies, and integrated real-time intrusion detection for future work [80]. Nazir et al. extended protection to trading closure and settlement integrity, but their SMPC-based design incurs higher communication overhead as participant scale increases and does not address storage elements, line losses, smart-contract vulnerabilities, replay attacks, or front running [65]. Evaluation therefore needs to account for protocol compatibility, device heterogeneity, topological dynamics, latency, and communication cost.

Authentication and access-control mechanisms improve session establishment, device legitimacy checking, key management, and runtime spoofing or anomaly detection [60,61,62]. Their deployment depends on key distribution, credential renewal, firmware or gateway support, local calibration, and false-positive handling. Current mechanisms protect individual transmission and admission stages but provide limited guidance on record admissibility during downstream trading reuse. Table 9 summarizes the framework-specific impacts, representative solutions, and remaining limitations.

### 5.3. Mitigating Confidentiality and Privacy-Exposure Risks

The third mitigation area addresses the confidentiality and privacy risks discussed in Section 4.3: reducing the visibility of fine-grained meter data and limiting the linkage of participants, bids, trades, billing records, and settlement artifacts across repeated trading stages. In the data-handling layer, van Schendel and Varenhorst proposed a time-aggregation approach for household energy profiles in which the temporal resolution of smart-meter data is reduced so that fine-grained behavioral signatures become less identifiable. Their results show that this brings privacy benefits and makes behavior more difficult to identify, including in an electric vehicle (EV)-charging example, although it also significantly affects demand-side management performance [82]. More broadly, recent surveys of privacy-preserving data aggregation in smart grids indicate that aggregation remains a major technical direction with respect to reducing the direct exposure of individual household data while still supporting system-level computation [83]. In a more trading-specific setting, Alàs and Sebé proposed a privacy-preserving electricity trading system for connected microgrids in which the DSO can compute the quantity to be charged or paid to each household at the end of a billing period without tracing that household’s detailed consumption profile [36]. These mechanisms reduce fine-grained privacy risk by lowering temporal granularity or limiting visible participant-level detail before trading reuse.

Reducing fine-grained visibility addresses only part of the privacy problem because privacy loss in energy trading can also arise when participant identities, bids, transactions, and settlement records become linkable across stages. A second group of studies addresses this linkage problem through privacy-preserving authentication, encrypted market clearing, encrypted smart-contract execution, and privacy-preserving billing.

In the authentication layer, Son et al. proposed a privacy-preserving authentication scheme for blockchain-based energy trading. The scheme uses lightweight attribute-based encryption to support access control and matching between energy users and sellers while completing mutual authentication and key agreement without the participation of an energy broker [38]. In the market-clearing layer, Wang et al. designed a three-layer blockchain-based P2P trading architecture in which homomorphic encryption and secure multi-party computation (SMPC) protect bidding-price and quantity information during encrypted order ranking and clearance [40]. Mitrea et al. addressed the smart-contract layer by encrypting transactional energy data with partial homomorphic encryption, allowing trading and settlement functions to operate over ciphertext [50].

For local energy markets, Erdayandi and Mustafa proposed Privacy-Preserving Local Energy Market (PP-LEM), which combines Stackelberg game-based clearance with a partially homomorphic cryptosystem and supports privacy-preserving market clearance for 200 users within the order of seconds [51]. For later billing and settlement, Erdayandi et al. proposed Privacy-Preserving and Accountable Billing (PA-Bill), a framework that combines homomorphic encryption, blockchain-based accountability, universal cost splitting, and dispute resolution. PA-Bill supports accurate billing of discrepancies between committed and delivered energy volumes without sacrificing privacy, with evaluations covering communities of up to 2000 households [18]. These methods reduce linkage- and identity-related privacy risks by redesigning authentication, clearance, billing, and settlement so that repeated market interactions expose less linkable participant information.

These mechanisms reduce privacy exposure at protected stages, but their limitations depend on later record reuse. Time aggregation lowers identifiability by reducing temporal resolution, but it also reduces operational detail [82]. Aggregation-oriented methods mainly protect data handling and provide limited coverage of later trading-stage linkage [83].

For trade linkage and identity disclosure, current mechanisms protect different workflow stages. Privacy-preserving authentication reduces broker-mediated or participant-level association during login and key establishment [38]. Encrypted trading strategies protect bid price and quantity during on-chain market clearance [40], while encrypted smart-contract mechanisms protect transactional data during contract execution and settlement logic [50]. PP-LEM supports privacy-preserving clearance for local markets at the scale of 200 users [51], and PA-Bill supports discrepancy-aware billing for communities of up to 2000 households [18].

Scalability depends on more than user count. Clearing frequency, transaction volume, participant churn, network constraints, billing workload, dispute workload, and settlement frequency also shape practical feasibility. Homomorphic encryption, SMPC, encrypted bidding, and blockchain-assisted billing reduce disclosure at protected stages but may add computation, communication, storage, and audit burden. Linkage risk can reappear when records are reused for accountability, dispute resolution, or settlement correction. Table 10 summarizes these framework-specific impacts, mitigation directions, and remaining limitations.

The framework-specific tables above summarize mitigation directions and remaining limitations across issue layers. To complement these qualitative comparisons, Table 11 reports quantitative and deployment-oriented indicators from representative mitigation studies where such information is available. The values are not used to rank the methods directly because the studies differ in attack models, datasets, system settings, and evaluation objectives.

## 6. Future Work

Existing mechanisms protect record formation, transmission, interface admission, privacy-sensitive reuse, billing, and settlement at individual stages of the trading workflow. Future research should connect these protections across record admission, temporal interpretation, disclosure, correction, and reuse after records cross technical and organizational boundaries. Four directions require further attention.

### 6.1. Post-Challenge Governance and Record Admissibility

Methods for tamper detection, false-data injection detection, message-order protection, Byzantine resilience, and accountable billing can identify challenged records or discrepant outcomes [17,18,58,64,69]. Trading workflows also require explicit decisions on the later use of these records. Future work should:

- Define admissibility statuses for challenged records, such as accepted, corrected, downgraded, conditionally accepted, rejected, or audit-only;
- Evaluate these statuses in cases involving bypass tampering, false-data injection, replayed or delayed interval records, and committed-versus-delivered energy mismatches.

### 6.2. Temporal and Operational Continuity Across Trading Stages

Ordering and synchronization protections improve the reliability of time-dependent exchanges [17,68,78]. Later trading stages also require a consistent timing history for each record. Future work should:

- Define interval-level metadata comprising the measurement interval, creation time, receipt time, sequence number, synchronization status, and record lineage;
- Measure the interval-misattribution rate; ordering-error rate; late-record handling time; and settlement-window mismatch under replay, replacement, reordering, delay, and clock drift.

### 6.3. Workflow-Wide Privacy–Accountability Co-Design

Privacy mechanisms protect aggregation, clearance, authentication, smart-contract execution, and billing at specific stages [18,38,40,50,82]. Cross-stage reuse can link records, bids, billing artifacts, and settlement outcomes, while disputes may require controlled disclosure [19]. Future work should:

- Specify which record fields are visible to market operators, validators, billing entities, settlement platforms, participants, and dispute arbiters at each trading stage;
- Design bounded-disclosure mechanisms for audit, committed-versus-delivered correction, dispute resolution, and settlement adjustment, evaluated through linkage risk, re-identification risk, disclosure size, and billing accuracy.

### 6.4. Deployable Protection Across Hybrid Trading Environments

Deployment studies demonstrate the relevance of transactive implementations, secure AMI communication, blockchain-supported TE/P2P frameworks, decentralized P2P–TES implementation, and network-secure P2P operation [29,43,55,80,84]. Future work should:

- Build mixed testbeds connecting smart meters, gateways, AMI communication paths, local controllers, market platforms, blockchain or audit layers, and billing or settlement modules;
- Benchmark protection mechanisms under heterogeneous devices, topology changes, intermittent connectivity, participant churn, short clearing intervals, and mixed centralized–transactive–P2P workflows;
- Evaluate deployment using latency, message overhead, validation accuracy, settlement correctness, interoperability, and deployment effort.

These directions target lifecycle-level protection of smart meter-derived records across practical energy-trading workflows.

## 7. Conclusions

This review examined cybersecurity issues in smart meter-based energy trading through the lifecycle of smart meter-derived records. Treating these records as the common analytical object links smart-meter sensing, communication, trading architecture, validation, billing, settlement, and privacy exposure within a single framework. This perspective shows that a meter-originated record does not carry a fixed risk profile; its trust requirements change as it moves from source-side measurement to communication, coordination, validation, billing, and settlement. The comparison of centralized, transactive, and peer-to-peer trading shows that architecture matters because each framework handles these records under different trust conditions. Existing countermeasures already provide important stage-level protection, including tamper and false-data detection, secure transmission, authentication, encrypted market clearance, and privacy-preserving billing. The main limitation is the lack of continuity across these protected stages. Record admissibility, temporal validity, privacy boundaries, and settlement relevance can weaken when records become delayed, challenged, partially disclosed, disputed, or reused across heterogeneous trading workflows. Future cybersecurity assessment for smart meter-based energy trading should focus on lifecycle-aware assurance of smart meter-derived records, especially in hybrid edge-to-market environments where records are handled across multiple actors and trust domains.

## Figures and Tables

**Table 1:** Comparison of representative existing reviews and the present review.

Review	Main Research Object	Analytical Dimensions	Trading Architecture Coverage	Security Issue Classification	Future Research Focus
Faia et al. [5]	Local electricity markets	Market benefits, barriers, current trends, and implementation perspectives	Covers local market designs and participation models	Security and privacy are not used as the main classification basis	Market development, regulation, participation, and implementation barriers
Gorbatcheva et al. [7]	P2P energy trading, transactive energy, and community self-consumption	Conceptual definitions and distinguishing characteristics	Explicitly compares P2P trading, transactive energy, and community self-consumption	Cybersecurity issues are not organized around trading-stage record handling	Clearer terminology, conceptual boundaries, and community energy design
Tooki and Popoola [8]	Transactive energy systems	Concepts, models, metrics, technologies, challenges, and policy issues	Focused mainly on transactive energy	Cybersecurity is discussed as one challenge area, without a record lifecycle classification	Transactive energy models, implementation challenges, enabling technologies, and policy directions
Tanis et al. [9]	Peer-to-peer energy trading	Market structure, operational layers, energy cooperatives, and multi-energy systems	Focused mainly on P2P trading and related operational layers	Security and privacy are discussed as implementation challenges, without lifecycle issue-layer classification	P2P market operation, scalability, regulation, and multi-energy integration
Athanasiadis et al. [2]	Smart-meter data analytics	Distribution-network applications, data analytics methods, and data-driven operation	Does not focus on energy trading architectures	Cybersecurity is not organized around trading-stage record risks	Smart-meter analytics for distribution-network operation, planning, and control
Ajiboye et al. [11] and Bibi et al. [13]	AMI data, smart-grid privacy, and privacy-preserving technologies	AMI security, privacy risks, cryptographic methods, and privacy-preserving mechanisms	Energy trading architectures are not the primary comparison frame	Focuses on AMI privacy and security mechanisms, with limited attention to record reuse across trading stages	Privacy-preserving technologies, secure AMI communication, and smart-grid data protection
This review	Smart meter-derived records in energy trading	Record formation, transmission, interface admission, validation, billing, settlement, and audit-related reuse	Compares centralized trading, transactive energy, and P2P trading as the main record-handling and trust settings, with hybrid or multi-framework studies discussed separately	Classifies issues into record integrity and temporal consistency, insecure transmission and interface access, and confidentiality and privacy exposure	Lifecycle-level record admissibility, temporal continuity, privacy–accountability co-design, and deployable protection in hybrid trading environments

**Table 2:** Analytical lifecycle forms of smart meter-derived records used in this review.

Lifecycle Form	Meaning in This Review	Typical Trading Use	Main Security Relevance
Raw metering data	Time-referenced measurements produced at or near the smart meter, including consumption, generation, import, export, and interval readings [1,16].	Provide the source evidence for later trading, aggregation, reporting, or verification.	Exposed to sensing-path tampering, meter bypass, local manipulation, timestamp errors, and clock desynchronization.
Trading input data	Meter-derived values or abstractions submitted into coordination, bidding, matching, or local market processes [7,9].	Support participant offers, local surplus or deficit estimation, flexibility coordination, and market interaction.	Affected by false data injection, replay, delayed submission, unauthorized modification, and strategic misreporting.
Validation data	Records, metadata, signatures, anomaly scores, timestamps, or consistency checks used to assess whether a submitted trading input is trustworthy [17,20].	Support authenticity checking, integrity verification, temporal checking, and delivery-consistency assessment.	Depend on authentication, provenance, synchronization, interface admission, and anomaly-detection reliability.
Settlement data	Validated or corrected records used for billing, payment, committed-versus-delivered reconciliation, or settlement adjustment [18,21].	Determine financial outcomes, settlement quantities, billing corrections, and participant obligations.	Sensitive to temporal misattribution, disputed delivery, incorrect correction, privacy leakage, and settlement manipulation.
Audit records	Retained evidence used after the trading cycle for dispute resolution, accountability checking, privacy-violation review, or later record verification [18,19].	Support later review of challenged transactions, billing disputes, privacy claims, and settlement decisions.	Require integrity preservation, controlled disclosure, traceability, and privacy-aware accountability.

**Table 3:** Comparison of energy trading architectures from the perspective of smart meter-derived records and cybersecurity-relevant workflow dimensions.

Attribute	Centralized	Transactive Energy	Peer-to-Peer
Data-path structure	Predominantly platform-facing and relatively bounded [6,24]	Coordinated multi-step flow across local and higher-layer entities [8,26]	Participant-facing and multi-hop across distributed trading components [9,29]
Coordination pattern	Central platform or aggregator control [6,24]	Iterative coordination with repeated feedback and state exchange [26,30]	Direct or platform-mediated participant interaction and matching [9,31]
Trust structure	Relatively concentrated under a dominant platform or operator [6,23]	Partially distributed across local control and coordination layers [7,8]	More fragmented across gateways, platforms, validators, and participants [7,29]
Role of smart meter-derived records	Billing, aggregation, centralized coordination, and settlement input [6,32]	Local state input, coordination support, and market interaction input [26,30]	Matching, transaction logic, validation, and settlement-related input [18,33]
Validation/settlement positioning	Mainly retained within platform-centered processing [6,32]	Distributed across coordination and market-facing layers [17,26]	More participant-facing and increasingly reliant on distributed validation or settlement support [18,29]
Key interfaces and attack surfaces	Meter–gateway–platform or aggregator-facing interfaces; exposure at the metering source, gateway transmission, platform ingestion, and centralized validation pipeline [11,34]	Meter–controller–gateway–coordinator–market interfaces; exposure at repeated coordination links, local controllers, gateways, and market-facing exchanges [14,17]	Participant–gateway–platform, peer-facing, blockchain, or smart-contract interfaces; exposure at participant access points, peer exchange, distributed validation layers, and settlement logic [29,35]
Scope of data exposure	Concentrated visibility within utility, aggregator, distribution-system operator, or platform-side processing [12,36]	Cross-layer visibility across controllers, coordinators, gateways, and market-facing entities [17,37]	Wider exposure across participants, platforms, validators, blockchain records, billing artifacts, and settlement outputs [38,39]
Validation responsibility	Central platform, aggregator, utility, distribution-system operator, or market operator [6,32]	Coordinators, local controllers, aggregators, or market-facing coordination layers [26,30]	Platform operator, peers, validators, smart contracts, blockchain-supported mechanisms, or hybrid arrangements [18,29]
Privacy and settlement-dispute risks	Centralized correlation of metering, billing, and account records; disputes may arise from aggregation, validation, billing, or operator-side settlement errors [34,36]	Repeated reuse of local state information and participant responses; disputes may arise from mistimed records, inconsistent coordination, scheduling errors, or settlement-window interpretation [17,21]	Linkage among participant identities, bids, transactions, smart-contract execution, billing records, and settlement outcomes; disputes may arise from committed-versus-delivered mismatch, peer transaction disagreement, or distributed reconciliation burden [18,40]

**Table 4:** Comparison of traditional centralized energy trading studies and their main architectural features.

Paper	Central Platform/ Coordinator	Platform-Facing Workflow	Aggregator/ Community Manager Explicit	Auction/Clearing Explicit	User/Prosumer Preference Explicit	Billing/Settlement/ Post-Delivery Explicit
[6]	✓	✓	×	×	×	×
[23]	✓	✓	×	✓	✓	×
[24]	✓	✓	✓	×	×	×
[41]	✓	✓	×	✓	×	×
[32]	✓	✓	×	×	×	✓
[42]	✓	✓	✓	×	×	✓

*Note:* A ✓ indicates that the feature is explicitly addressed in the selected study; × indicates that the feature is not explicit or not reported.

**Table 5:** Comparison of transactive energy studies with emphasis on local measurement inputs, distributed coordination, and deployment-relevant interaction.

Paper	Local Measurement/ Prosumer State Input Explicit	Local Decision/ Coordination Layer Explicit	Repeated/ Iterative Coordination Explicit	Trading/ Price Signal Explicit	Hierarchical Market Interface/ Coordinator Explicit	Deployment/ Interoperability Explicit
[26]	✓	✓	✓	✓	✓	×
[17]	×	×	✓	×	✓	×
[44]	×	✓	✓	✓	×	×
[30]	✓	✓	✓	✓	×	×
[37]	✓	×	✓	✓	×	×
[43]	×	×	✓	×	✓	✓

*Note:* A ✓ indicates that the feature is explicitly addressed in the selected study; × indicates that the feature is not explicit or not reported.

**Table 6:** Comparison of peer-to-peer energy trading studies and their main architectural features.

Paper	Participant-Side Local Input Explicit	Peer Matching/ Market Clearing Explicit	Participant-Facing Transaction Logic Explicit	Blockchain/ Smart-Contract/ Distributed Validation Explicit	Privacy-Preserving Mechanism Explicit	Settlement/ Post-Trade Accountability Explicit
[33]	✓	×	✓	×	✓	×
[49]	✓	✓	✓	×	×	×
[40]	✓	✓	✓	✓	✓	✓
[50]	✓	×	✓	✓	✓	✓
[51]	✓	✓	✓	×	✓	×
[18]	✓	×	×	✓	✓	✓

*Note:* A ✓ indicates that the feature is explicitly addressed in the selected study; × indicates that the feature is not explicit or not reported.

**Table 7:** Workflow-based attacker capabilities across smart meter-derived record lifecycle forms.

Attacker Type	Assumed Capability	Affected Lifecycle Form	Main Security Consequence
Meter-side attacker	Manipulates the smart meter, sensing path, local measurement process, timestamp source, local gateway, or local reporting path [57,58].	Raw metering data and early trading input data.	False or mistimed records may be generated before validation, billing, or settlement reuse.
Communication-path attacker	Intercepts, modifies, delays, drops, replays, replaces, or reorders records and trading messages between meters, gateways, controllers, coordinators, platforms, or peers [17,59].	Trading input data and validation data in transit.	Records may arrive altered, duplicated, stale, out of order, or detached from their intended reporting or settlement interval.
Interface or credential attacker	Uses spoofed identities, compromised credentials, weak authentication, or illegitimate session establishment to submit records, requests, commands, or trading messages [60,61,62].	Interface-admitted trading input data and validation data.	Untrusted inputs may be accepted as legitimate, affecting coordination, validation, billing, or settlement decisions.
Malicious or strategic participant	Submits manipulated bids; false local-state information; inaccurate delivery claims, false data; or manipulated import, export, generation, or consumption values [63,64,65].	Trading input data, validation evidence, and settlement data.	Matching, pricing, delivery reconciliation, and participant obligations may be distorted, especially in participant-facing workflows.
Privacy or linkage adversary	Observes, correlates, or links fine-grained records, bids, transactions, participant identifiers, billing artifacts, or settlement outcomes across repeated trading stages [13,38,39,40].	Trading input data, settlement data, and audit records.	Household behavior, participant identity, bidding behavior, or transaction history may become inferable across trading workflows.
Settlement or audit-stage attacker	Affects validation evidence, settlement inputs, smart-contract execution, billing correction, or dispute-related records [18,35,50].	Settlement data and audit records.	Billing correction, committed-versus-delivered reconciliation, dispute handling, and settlement accountability may become unreliable.

**Table 8:** Framework-specific impacts, representative solutions, and limitations for record integrity and temporal consistency in smart meter-based energy trading.

Issue	Traditional Centralized Trading	Transactive Energy	Peer-to-Peer Trading	Representative Solutions	Remaining Limitation
**Record integrity**	Distorted aggregation, validation, and settlement preparation [67,69].	Falsified-record propagation through iterative coordination loops [17,70].	Malicious data and Byzantine manipulation [64]; disputed billing and reconciliation pressure [18,65].	Embedded load injection [58]; FDIA detection/correction in local energy networks [69]; load-aggregator FDIA detection [67]; metering-platform anomaly detection [77]; Byzantine-resilient anomaly detection in P2P trading [64].	Targeted protection at multiple attack points; deployment depends, where applicable, on meter-side support, training data, and near-real-time processing; limited basis for later admissibility and reuse decisions.
**Temporal consistency**	Stale or mistimed records disrupt validation and settlement timing [21,66].	Replay, replacement, and reordering degrade coordination [17]; desynchronization affects scheduling reliability [66,68].	Timing and delivered-volume mismatches increase validation, billing, and settlement-reconciliation pressure after multiple handoff points [18].	TES protection with signatures and stamp concatenation [17]; synchronization-targeted false-data detection [68]; lightweight IEEE 1588 PTP attack detection and mitigation [78].	Protection remains split across timing layers; synchronization and latency requirements may affect deployment; limited continuity of later temporal validity.

**Table 9:** Framework-specific impacts, representative solutions, and limitations for insecure transmission and unauthenticated or unauthorized access at trading interfaces in smart meter-based energy trading.

Issue	Traditional Centralized Trading	Transactive Energy	Peer-to-Peer Trading	Representative Solutions	Remaining Limitation
**Insecure transmission**	Platform-facing local energy market workflows [6]; compromised data ingestion and market/settlement preparation under cyber attacks [34].	TE coordination is exposed to communication-layer attacks [14]; replay, replacement, and reordering can propagate through iterative coordination [17,72].	Participant-facing and blockchain-enabled P2P workflows create fragmented communication paths [9,29]; secure P2P pricing designs highlight tampering-related verification and settlement-integrity pressure [65].	OSCORE-over-6TiSCH end-to-end protection [80]; lightweight hybrid signcryption [81]; additive secret sharing, MAC verification, and blockchain-enabled P2P settlement [65].	Protocol compatibility, topological dynamics, device constraints, participant-scale communication overhead, and settlement-layer coverage remain practical constraints [65,80].
**Unauthenticated or unauthorized access at trading interfaces**	Bounded platform-facing validation chain [6]; unauthorized admission risk under weak AMI authentication or intrusion detection [60].	Untrusted inputs are injected into controller–gateway–coordinator loops and distort higher-layer decisions [14,72].	Participant-facing and blockchain-enabled P2P workflows create multi-hop exchange before distributed validation and settlement closure [9,29]; participant authentication increases identity-verification requirements [38]; untrusted-input risks increase settlement-accountability pressure [65].	Anonymous, certificateless authentication and key agreement [61]; ECC/PUF/ blockchain-assisted authentication and key agreement [62]; IAIDS with RSSI-aware anomaly detection [60].	Protocol-level authentication, device legitimacy checking, and AMI runtime detection remain separate; key management, credential renewal and revocation, calibration, and cross-interface authorization remain practical constraints [60,61,62].

**Table 10:** Framework-specific impacts, representative solutions, and limitations for confidentiality and privacy exposure of trading-relevant meter data in smart meter-based energy trading.

Issue	Traditional Centralized Trading	Transactive Energy	Peer-to-Peer Trading	Representative Solutions	Remaining Limitation
**Fine-grained meter-data exposure and behavioral inference**	Smart-meter data can expose household production and consumption patterns [12]; connected microgrid trading shows the need to limit operator-side visibility [36].	Repeated TE coordination reuses local measurements across controllers and market interactions [17]; privacy-preserving coordination work highlights cross-layer privacy concerns [37].	Participant-facing P2P workflows increase exposure of meter-derived information [9,29]; socio-demographic inference from smart-meter readings shows how prosumer behavior can become market-relevant [49].	Time aggregation for household energy profiles [82]; privacy-preserving data aggregation [83]; controlled-disclosure trading and billing in connected microgrids [36].	Time aggregation creates a privacy–operational detail trade-off; aggregation mainly protects data handling and gives limited coverage of later trading-stage linkage [82,83].
**Trade linkage and identity disclosure**	Metering, billing, and account records remain correlatable under a single authority [36].	Repeated coordination creates more opportunities for cross-stage linkage of local measurements and participant responses [17,37].	Participant authentication and repeated trading create association risks [38]; bidding information can be exposed during clearance [40]; on-chain transactional and smart-contract data create linkage risks [39,50]; billing artifacts reinforce linkage over time [18].	Privacy-preserving authentication [38]; encrypted bidding [40]; privacy-preserving clearance [51]; encrypted smart-contract execution [50]; privacy-preserving billing [18].	Protection remains stage-specific; linkage risk can reappear across authentication, clearance, smart-contract execution, billing, and settlement, while overhead, clearing frequency, billing workload, and controlled disclosure shape scalability and deployment feasibility.

**Table 11:** Reported quantitative and deployment-oriented indicators in representative mitigation studies.

Study	Mitigation Focus	Reported Indicator	Evaluation Setting	Interpretation/Limitation
Molina-Moreno et al. [58]	Partial-bypass tamper detection	100% sensitivity and detection accuracy in 100 simulated cases; all tested bypass conditions were detected across four physical scenarios; estimated detection cycle below 2s	Hardware-assisted embedded-load injection in the smart-meter sensing path	Strong source-side tamper-detection result but based on limited physical scenarios; commercial firmware integration and environmental robustness remain to be explored future work.
Kermani et al. [69]	FDIA detection and correction in local energy-network trading	Maximum attack-effect mitigation accuracy of 91.67%	Simulation-based evaluation in interconnected local energy networks with energy and flexibility transactions	Provides a direct accuracy indicator for trading-related FDIA mitigation; results remain tied to the simulated network and attack setting.
Zhu et al. [77]	FDIA detection in electric-energy metering platforms	Detection accuracy above 99.97%; delay below 0.04 s; maximum packet capture rate of $7.1\times 10^{4}$ pps	4000 metering-data records and 1000 injected false-data samples in an automatic metering-data collection platform	Strong accuracy and latency indicators; evaluation remains platform-specific and requires further validation under more complex attack scenarios and highly concurrent settings.
Cheng et al. [61]	Anonymous, certificateless authentication and key agreement	Authentication transmission reduced from three messages to two messages; communication and computation cost compared	Protocol-level security and performance assessment for smart-grid authentication	Useful overhead indicator for authentication but does not evaluate trading-scale deployment or settlement-stage reuse.
Nazir et al. [65]	Secure P2P pricing, transmission verification, and blockchain settlement	Evaluation on a 4-year real-time dataset with 22 participants	Additive secret sharing, MAC verification, modified VAM allocation, and blockchain-enabled settlement	Provides dataset and participant-scale evidence for secure P2P trading; larger participant-scale communication overhead remains a key constraint.
Erdayandi and Mustafa [51]	Privacy-preserving local-market clearance	Market clearance for 200 users within the order of seconds	Partially homomorphic cryptosystem with Stackelberg game-based local-market clearance	Provides a clear runtime and user-scale indicator for privacy-preserving clearance; results depend on the clearance model and market assumptions.
Erdayandi et al. [18]	Privacy-preserving and accountable billing	Support for communities of up to 2000 households	Semi-decentralized billing with homomorphic encryption, blockchain accountability, and dispute resolution	Provides a scalability indicator for privacy-preserving billing; scope is billing and accountability, with limited coverage of the full trading workflow.

## Data Availability

No new data were created or analyzed in this study. Data sharing is not applicable to this article.
